# Neuronal Phenotype of *col4a1* and *col25a1*: An Intriguing Hypothesis in Vertebrates Brain Aging

**DOI:** 10.3390/ijms23031778

**Published:** 2022-02-04

**Authors:** Adele Leggieri, Chiara Attanasio, Antonio Palladino, Paolo de Girolamo, Carla Lucini, Livia D’Angelo

**Affiliations:** 1 Department Veterinary Medicine and Animal Production, University of Naples Federico II, Via F. Delpino 1, 80137 Naples, Italy; adele.leggieri@unina.it (A.L.); chiara.attanasio@unina.it (C.A.); degirola@unina.it (P.d.G.); carla.lucini@unina.it (C.L.); 2 Department Agricultural Sciences, University of Naples Federico II, Via Università 100, 80055 Portici, Italy; antonio.palladino@unina.it

**Keywords:** *Nothobranchius furzeri*, collagens, fish, central nervous system, aging markers

## Abstract

Collagens are the most abundant proteins in vertebrates and constitute the major components of the extracellular matrix. Collagens play an important and multifaceted role in the development and functioning of the nervous system and undergo structural remodeling and quantitative modifications during aging. Here, we investigated the age-dependent regulation of *col4a1* and *col25a1* in the brain of the short-lived vertebrate *Nothobranchius furzeri*, a powerful model organism for aging research due to its natural fast-aging process and further characterized typical hallmarks of brain aging in this species. We showed that *col4a1* and *col25a1* are relatively well conserved during vertebrate evolution, and their expression significantly increases in the brain of *N. furzeri* upon aging. Noteworthy, we report that both *col4a1* and *col25a1* are expressed in cells with a neuronal phenotype, unlike what has already been documented in mammalian brain, in which only *col25a1* is considered a neuronal marker, whereas *col4a1* seems to be expressed only in endothelial cells. Overall, our findings encourage further investigation on the role of *col4a1* and *col25a1* in the biology of the vertebrate brain as well as the onset of aging and neurodegenerative diseases.

## 1. Introduction

Aging is a progressive and irreversible process associated with physical and functional decline, and therefore considered the primary risk factor in the onset and exacerbation of neurodegenerative diseases [1,2].

According to Wyss-Coray (2016), it is possible that normal brain aging almost physiologically forms a continuum with neurodegeneration and disease given the high incidence of neurodegenerative diseases in the elderly and the rarity of disease-free brains with advancing age [3]. However, this author recognized that considering neurodegeneration, the natural expression of hastened aging is unhelpful as it is critical to understand how stochastic factors interact in defining a specific disease. Therefore, to enhance our understanding of the aging contribution to disease, we need to analyze how environmental and genetic factors cause the onset of a particular disease rather than another, and recognize the relevance of these processes in the disease itself [3]. From this perspective, interesting findings stemmed from Podtelezhnikov et al. (2011), who performed an extended analysis of age- and disease-related changes in gene expression in the brain of people affected by Alzheimer’s disease (AD) and those disease-free. In this work, the authors showed signatures of accelerated aging in a neuronal-gene expression module identifying four transcriptional biomarkers including numerous collagen genes [4].

Collagens are the most abundant proteins in the body of a vertebrate, where they play a key role in a wide range of biological functions such as tissue architecture, cell differentiation, adhesion, migration, and tissue repair [5]. Collagens are generally secreted and organized into fibrils or supramolecular structures of the extracellular matrix [5]. However, collagens also exist as transmembrane proteins expressed on the cellular membrane, where they act as surface receptors or adhesion molecules [5]. These types of collagens are known as membrane-associated collagens with interrupted triple helices (MACITs) or unconventional collagens, and play a crucial role in cell adhesion in a variety of tissues including the nervous system [6]. Therefore, in consideration of the common involvement in brain aging, we undertook this study to investigate the age-dependent expression of two evolutionary conserved collagen genes, collagen type IV. and type XXV. Collagen type IV α 1 chain (*COL4A1*) is highly conserved across species and falls among the major functional components of external lamina of many cell types. *COL4A1* regulates cell motility, proliferation, and differentiation [7], and is a crucial element of the external lamina of the choroid plexuses, pia mater, and capillaries of mouse brain [7,8]. Interestingly, Uspenskaia et al. (2004) linked an increased accumulation of collagen type IV in the external lamina of human cerebral microvessels to microvascular alterations contributing to the development of stroke and vascular dementia [9]. Furthermore, collagen type IV content notably increases in the cerebral microvessels of patients affected by AD in comparison to age-matched controls [10]. Moreover, collagen type IV also accumulated in the external lamina of cerebral cortical capillaries in both AD and Parkinson’s disease patients [11]. In zebrafish, collagen type IV is widely expressed in the developing central nervous system (CNS), where it contributes to control the axonogenesis of cerebellar granule cells [12] and lamination of synaptic connections from the retina to the optic tectum [13,14].

Collagen type XXV α 1 chain (*COL25A1*) is the latest member of MACITs/unconventional collagens [5]. In mammals, *Col25a1* is specifically expressed in neurons, while lower expression has been detected in heart, testis, and eye [15]. Loss of collagen XXV impairs intramuscular growth of motor axons leading to motor neuron apoptosis [16], suggesting that *Col25a1* is required for correct neuromuscular development and function [5,16]. Interestingly, *COL25A1* was first described as a component of senile plaques of AD brains [15]. In fact, *Col25a1* is also known as CLAC-P (collagen-like Alzheimer amyloid plaque component) as it encodes for CLAC, a soluble form binding amyloid-β fibrils, which are the primary constituents of AD senile plaques [17]. Furthermore, three SNPs within *COL25A1* gene have been associated with increased risk of AD in a Swedish population-based longitudinal study [18]. Moreover, overexpression of collagen type XXV in transgenic mice leads to an AD-like phenotype development and animals died instantly after birth due to respiratory failure caused by diaphragm denervation [19].

In light of the common involvement of *Col4a1* and *Col25a1* in brain aging and based on different mechanisms due to their diverse intrinsic nature, we investigated their expression in the brain of *Nothobranchius furzeri*, a model organism capable of recapitulating all the hallmarks of mammalian brain aging despite its very short life cycle [20].

The African turquoise killifish *N. furzeri* is the vertebrate with the shortest lifespan ever reported in captivity [21,22]. This unique feature is reflected in a very fast growth coupled with the relatively early expression of aging phenotypes at morphological, physiological [23,24], and behavioral levels [25]. Overall, *N. furzeri* shows typical teleost aging features enhanced by high incidence of age-dependent neoplasia, making this species a suitable model for studying the molecular mechanisms underlying the aging process [26]. Furthermore, aged killifish, similar to mammals, display distinctive aging hallmarks such as lipofuscin accumulation, age-dependent gliosis, rapid decay of adult neurogenesis, low regenerative abilities, high inflammatory reaction, and glial scarring [26,27]. Nonetheless, the African turquoise killifish also shows clustering of age-related genes in specific genomic regions and positive selection for longevity determinants [20]. Altogether, these features make *N. furzeri* a powerful tool for aging studies by linking basic to preclinical research, with the translational value of this model organism [28].

Exploiting our knowledge of *N. furzeri* and its above-mentioned points of strength [29], in this work, we report for the first time that (a) COL4A1 and COL25A1 show a discrete degree of evolutionary conservation among vertebrates; (b) *col4a1* and *col25a1* expression displays age-related level changes coherently with a wide neuroanatomical pattern of expression; (c) col4a1 and col25a1 mRNAs are expressed in neurons.

## 2. Results

### 2.1. Identification and Evolutionary History of col4a1 and col25a1

**Figure 1:**
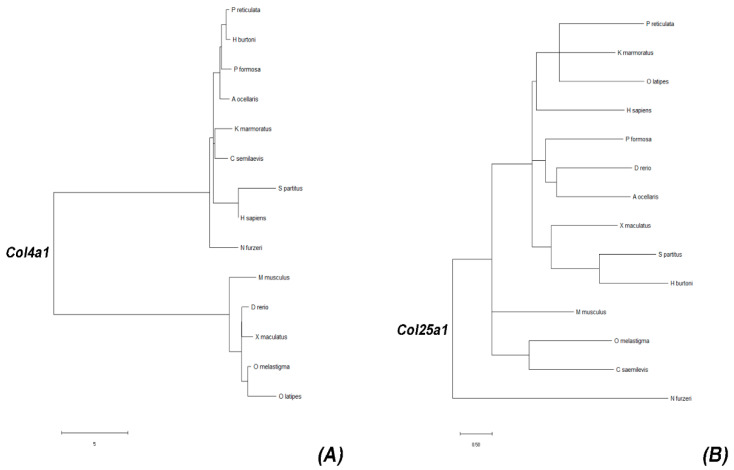
Gene tree representing *Col4a1* and *Col25a1* evolutionary history. (**A**) Branching pattern of *col4a1* of *N. furzeri* revealing a common ancestor with human COL4A1 in comparison to mice and zebrafish. (**B**) Branching pattern of *col25a1* of *N. furzeri* displaying the phylogenetic distance from the other selected species.

The branching pattern shows that *col4a1* of *N. furzeri* is closely related to most teleost fish species (Figure 1A). Interestingly, in this species, *col4a1* displays a common ancestor with human *COL4A1* in comparison to what happens in mice and zebrafish. Intriguingly, a clearly divergent evolutionary event occurred in the case of the *col25a1* branching pattern (Figure 1B), where *col25a1* in *N. furzeri* is phylogenetically distant from all the other selected species, being on a separated branch.

We also analyzed the degree of evolutionary conservation of COL4A1 and COL25A1 proteins by aligning sequences of human, mouse, zebrafish, and killifish for both proteins (Supplementary Figure S1). The aminoacidic sequence of *N. furzeri* col4a1 displayed 57.53% of identity with zebrafish, and 61.84% and 58.90% with human and mouse sequences, respectively. *N. furzeri* col25a1, instead, showed 72.62%, 58.17%, and 71.13% of identity with zebrafish, human, and mouse sequences, respectively.

### 2.2. Age-Related Expression of Brain Cell Lineage Markers and col4a1 and col25a1

Terzibasi Tozzini and colleagues [26] characterized, at the molecular level, the brain aging phenotype in *N. furzeri* and documented an increased level of some markers (*S100β*, *gfap*, and *pcna*) in 32-week old specimens compared to 25-week old ones. Therefore, we decided to characterize the brain phenotype in 27-week old *N. furzeri* to test whether brain aging is already well established at this age by measuring *S100β*, *gfap* and *pcna* [27], *dcx*, and *sox2* [30,31].

**Figure 2:**
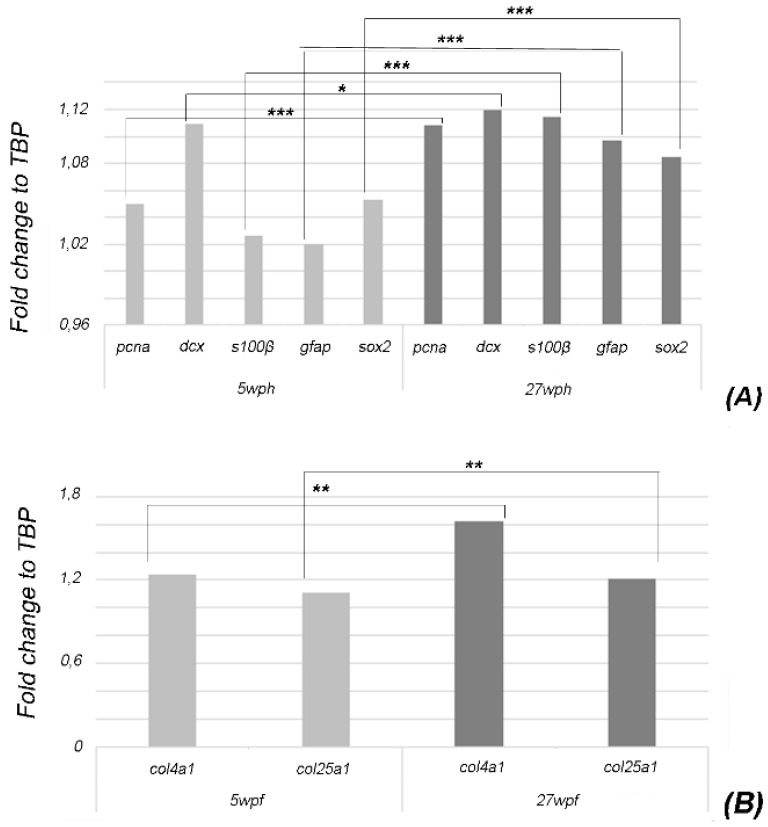
qPCR to evaluate the brain expression of pcna, dcx, sox2, s100β, gfap, col4a1, and col25a1 mRNAs in young (5wph) and old (27wph) animals. Expression levels were analyzed by the ΔΔCt method and normalized to the housekeeping gene TATA-box binding protein (TBP). The analysis was performed using the relative delta curve threshold (ΔΔCT) method. Graphic was built on fold change values to TBP. (**A**) Bar plot shows upregulation of *gfap*, *pcna*, *s100β*, and *sox2*. (**B**). Bar plot shows upregulation of *col4a1* and *col25a1* “*” indicates *p* ≤ 0.5, “**” indicates *p* ≤ 0.05, “***” indicates *p* ≤ 0.0001.

In compliance with Terzibasi Tozzini and colleagues (2012), our quantitative analyses showed significant upregulation for *gfap p* ≤ 0.0001, *s100β* (*p* ≤ 0.0001), and *pcna* (*p* ≤ 0.0001) over aging. Furthermore, we also observed a strong increase of *sox2* (*p* ≤ 0.0001) expression while a slight upregulation was detected for *dcx* (*p* ≤ 0.5) (Figure 2A).

We then measured the expression level of *col4a1* and *col25a1* in the brain of *N. furzeri* upon aging and, surprisingly, we observed a moderate upregulation (*col4a1: p* ≤ 0.05 and *col25a1: p* ≤ 0.05).

### 2.3. Neuroanatomical Localization of col4a1 and col25a1 mRNAs in the Brain of Young and Old Animals

#### 2.3.1. col4a1 and col25a1 mRNAs in the Brain of Young Animals

**Figure 3:**
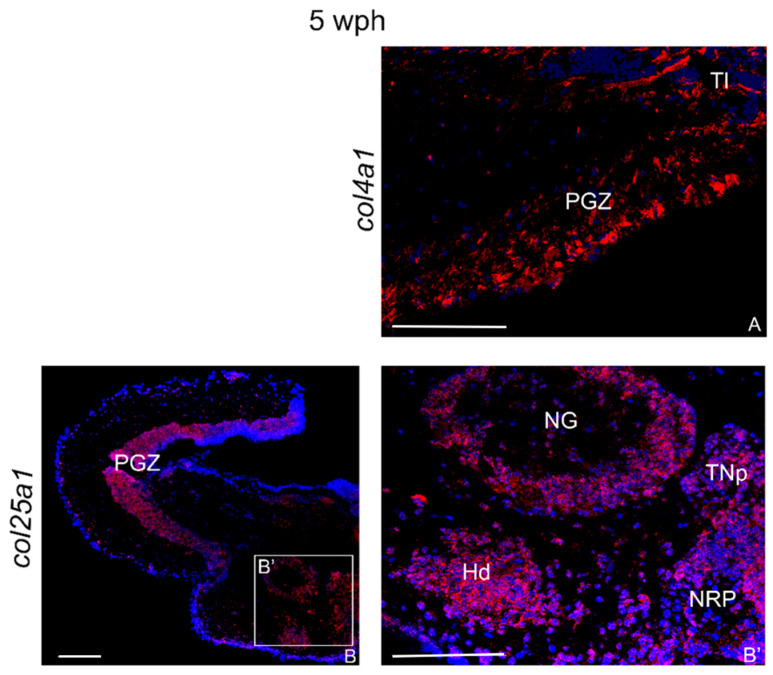
*col4a1* and *col25a1* mRNA distribution in transverse sections of the brains of young *N. furzeri*. (**A**) Optic tectum with intense staining in the periventricular gray zone and near the layer of the longitudinal tori. (**B**) Overview of diencephalon/midbrain with strong labeling in the optic tectum. (**B’**) Higher magnification of the inlet in (**B**) showing intense labeling of neurons in glomerular nucleus, nucleus of posterior recess, posterior tuberal nuclei, and dorsal hypothalamus. Abbreviations: Hd, dorsal hypothalamus; NG, glomerular nucleus; NRP, nucleus of the posterior recess; PGZ, periventricular gray zone; TNp, posterior tuberal nucleus. Scale bars: (**B**) 100 µm; (**A**,**B**’) 200 µm.

In the brain of young animals, col4a1 mRNAs appeared poorly expressed and restricted only to a few cells in the periventricular gray zone (PGZ) of the optic tectum (OT) and around the longitudinal tori (Tl) (Figure 3A). Conversely, col25a1 mRNA was abundantly expressed at the margin between caudal diencephalon and cranial midbrain, specifically in the glomerular nucleus (NG), dorsal hypothalamus (Hd), nucleus of posterior recess (NRP), and posterior tuberal nuclei (TNp) (Figure 3B,B’). In the midbrain, numerous positive cells were observed in the PGZ of the OT (Figure 3B).

#### 2.3.2. col4a1 mRNA in the Brain of Old Animals

The expression pattern of col4a1 mRNA in the brain of old animals was widely observed in all brain regions.

##### Forebrain

**Figure 4:**
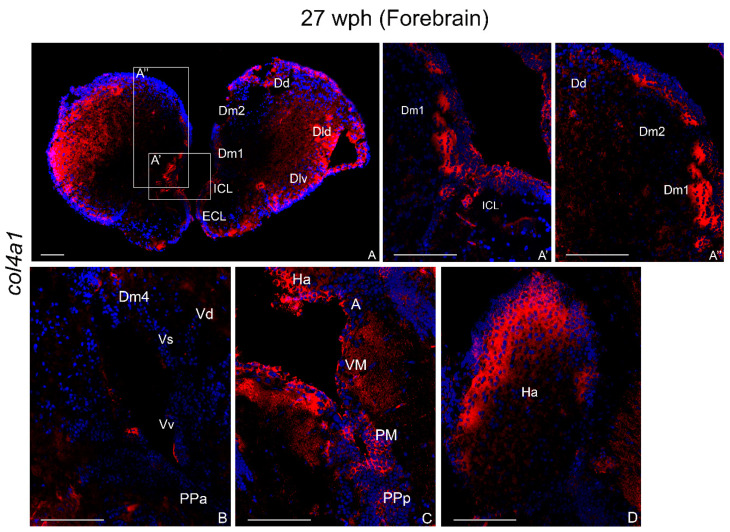
col4a1 mRNA distribution in transverse sections of forebrain of old *N. furzeri*. (**A**) Overview of the rostral telencephalon including olfactory bulbs. (**A’**) Higher magnifications of positive cells in the internal cellular layer of olfactory bulbs and in cell group 1 of the medial zone of dorsal telencephalon. (**A’’**) Higher magnifications of positive cells in cell groups 1–2 of medial zone and dorsal zone of dorsal telencephalon. (**B**) Expression in a few cells in cell group 4 of the medial zone of dorsal telencephalon, ventro-ventral zone of ventral telelencephalon, and in the anterior pre-optic area. (**C**) Expression in cells of habenular nucleus, thalamic nuclei, magnocellular, and posterior preoptic nuclei. (**D**) Numerous and intense expression in cells of habenular nucleus. Abbreviations: A, anterior thalamic nucleus; Dd, dorsal region of dorsal telencephalon; Dld, dorso-lateral region of dorsal telencephalon; Dlv, ventro-lateral region of dorsal telencephalon; Dm1-4, layers of the medial region of dorsal telencephalon; ECL, external cellular layer; Ha, habenular nucleus; ICL, internal cellular layer; PM, magnocellular pre-optic nucleus; PPa, anterior pre-optic nucleus; PPp, parvocellular portion of pre-optic nucleus; Vd, dorsal region of ventral telencephalon; VM, ventro-medial thalamic nucleus; Vs, supracommissural region of ventral telencephalon; Vv, ventral region of ventral telencephalon. Scale bars: (**A**) 100 µm; (**A’**,**A’’**,**B**,**C**,**D**) 200 µm.

col4a1 mRNA labeling was detected in dense and positive cells in the external (ECL) and internal cellular layer (ICL) of the olfactory bulbs (Figure 4A,A’). In the medial zone of dorsal telencephalon, few packed cells were observed in cell groups 1 and 2 (Dm1-2) (Figure 4A–A’’) and caudally in 4 (Figure 4B). Numerous cells were detected in the dorso-lateral zone (Dld) (Figure 4A). Few cells were labeled along the dorsal zone (Dd) and in the zones of ventral telelencephalon (Vd, Vv, Vp, and Vs) (Figure 4B). Strong probe signal was displayed along the telencephalic ventricles.

**Figure 5:**
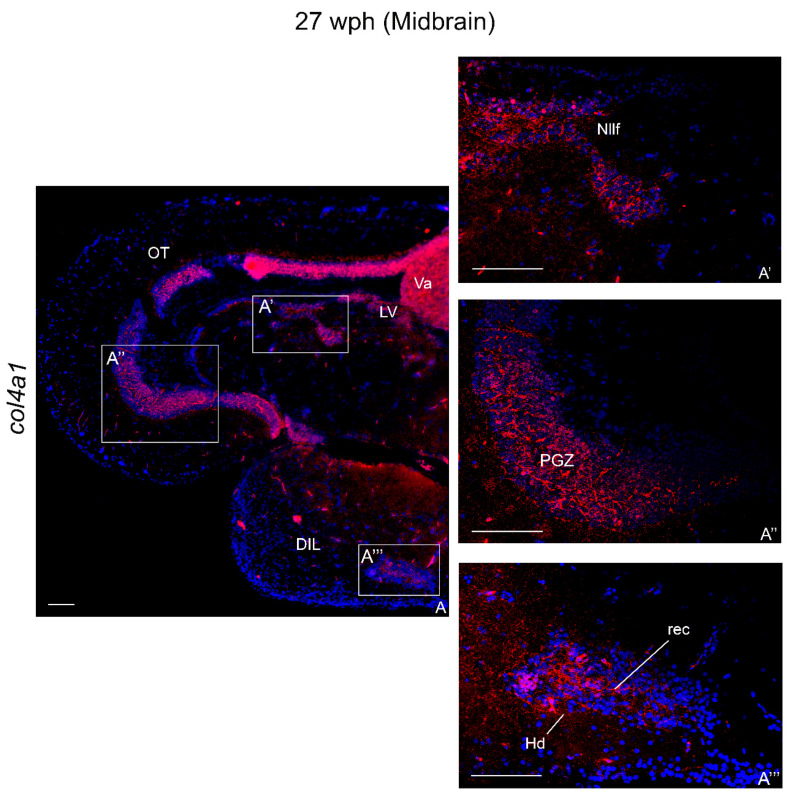
col4a1 mRNA distribution in transverse section of midbrain of old *N. furzeri*. (**A**) Overview of the midbrain. (**A’**) Strong expression in the nucleus of lateral longitudinal fascicle. (**A’’**) Strong expression in the periventricular gray zone of the optic tectum. (**A’’’**) Higher magnification of positive cells in the dorsal and inferior lobe of hypothalamus. Abbreviations: DIL, diffuse inferior lobe of hypothalamus; Hd, dorsal hypothalamus; Nllf, nucleus of lateral longitudinal fascicle; LV, nucleus of lateral valvula; OT, optic tectum; PGZ, periventricular gray zone; Tl, longitudinal tori; rec, hypothalamic recess; Va, valvula of cerebellum. Scale bars: (**A**) 50 µm; (**A’**,**A’**’,**A’’’**) 200 µm.

In the diencephalon, an intense col4a1 mRNA signal was detected in cells of the anterior preoptic (PPa) (Figure 4B) and posterior preoptic (PPp) nuclei, magnocellular preoptic (PM), ventro-medial (VM), and anterior (A) thalamic nuclei (Figure 4C) and habenular nuclei (Ha) (Figure 4C,D). More caudally, numerous intensely labeled cells in the dorsal hypothalamus and a few diffused cells in the inferior lobe of the hypothalamus were detected (Figure 5A,A’’’).

##### Midbrain

In the tegmentum, dense positive cells were observed nearby the nucleus of the lateral valvula (LV) (Figure 5A) and nucleus of the lateral longitudinal fascicle (Nllf) (Figure 5A,A’). col4a1 mRNA was detected in the PGZ (Figure 5A–A’’). An intense signal was observed in the Tl at the margin with the PGZ. Strong col4a1 mRNA signal was also displayed caudally in the layer of the semicircular tori.

##### Hindbrain

In the cerebellum, strong signal was observed in the valvula and in the granular and molecular layers of the body and, caudally, in the granular eminentiae. Several cells containing col4a1 mRNA were seen in the central griseum, at the dorsal margin with the medulla oblongata.

#### 2.3.3. col25a1 mRNA in the Brain of Old Animals

##### Forebrain

**Figure 6:**
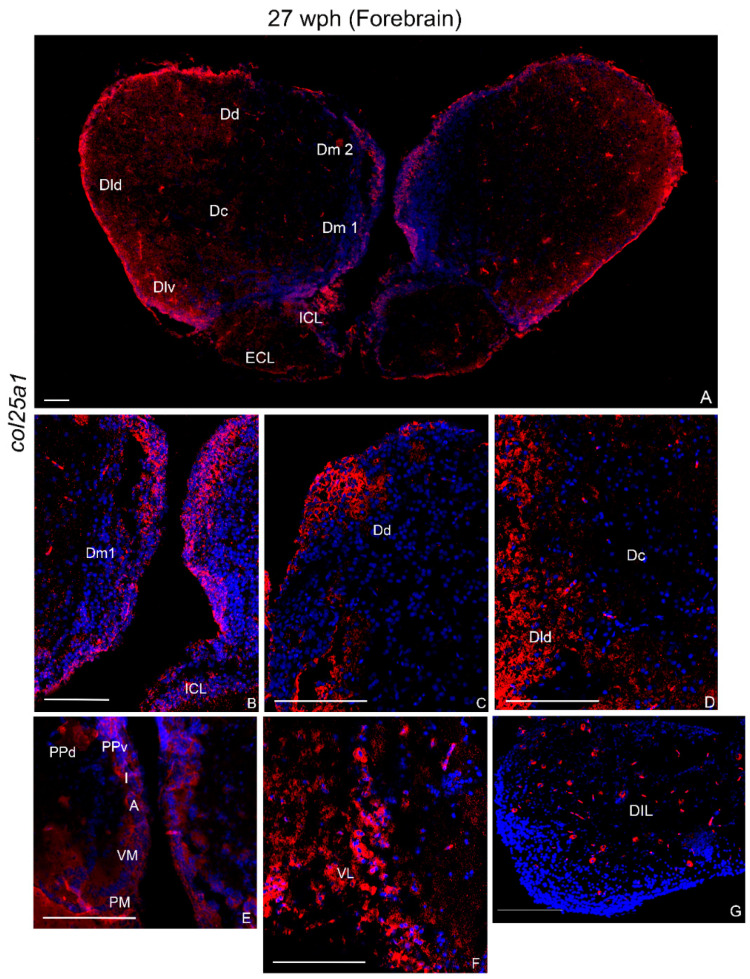
col25a1 mRNA distribution in transverse section of old *N. furzeri* forebrain. (**A**) Overview of labeling in the telencephalon and olfactory bulbs. (**B**–**D**) Higher magnification of positive cells in the cell groups 1 of medial, dorsal and central zones of dorsal telencephalon. (**E**) Intense expression in the cells of diencephalic periventricular pre-tectal and thalamic nuclei. (**F**) Labeling in cells of the ventro-lateral thalamic nucleus. (**G**) Labeling in sparse cells of the diffuse inferior lobe of the hypothalamus. Abbreviations: A, anterior thalamic nucleus; Dc, central zone of dorsal telencephalon; Dd, dorsal zone of dorsal telencephalon; Dld, dorso-lateral zone of dorsal telencephalon; Dlv, ventro-lateral zone of dorsal telencephalon; Dm1-2, layers of the medial zone of dorsal telencephalon; ECL, external cellular layer; I, inferior thalamic nucleus; ICL, internal cellular layer; PM, magnocellular pre-optic nucleus; PPd, dorsal periventricular pre-tectal nucleus; PPv, ventral periventricular pre-tectal nucleus; VM, ventro-medial thalamic nucleus; VL, ventro-lateral thalamic nucleus; Vs, supracommissural zone of ventral telencephalon; Vv, ventral zone of ventral telencephalon. Scale bars: (**A**) 100 µm; (**B**) 200 µm; (**C**–**G**) 300 µm.

In the telencephalon, col25a1 mRNA was detected in numerous positive cells in the internal cellular layer (ICL) of olfactory bulbs, mostly along the ventricular region and in the external cellular layer (ECL) (Figure 6A). Densely packed cells were observed in layers 1–2 of the medial zone of dorsal telencephalon (Figure 6A,B). Additionally, in the dorsal (Dd), lateral-dorsal (Dld), and latero-ventral (Dlv) (Figure 6A,C,D) zones of the dorsal telencephalon, numerous intensely positive cells were seen. In the ventral telencephalon, intense staining was detected in densely packed cells of the ventral (Vv) and supracommissural (Vs) zones.

**Figure 7:**
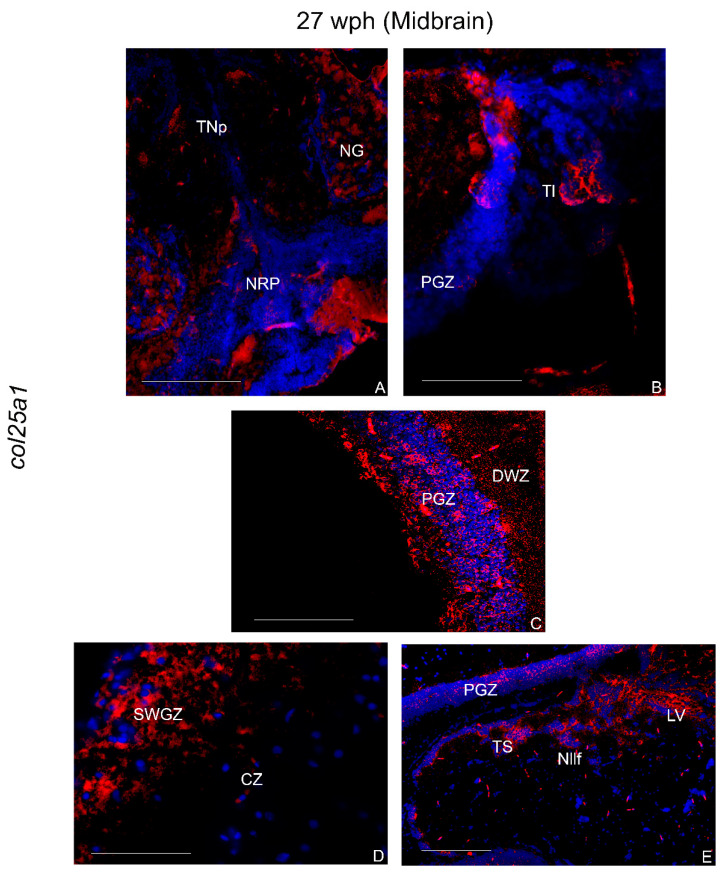
col25a1 mRNA distribution in transverse section of caudal diencephalon and mesencephalon of old *N. furzeri*. (**A**) Diffuse positive cells in the glomerular nucleus, nucleus of posterior recess, and posterior tuberal nucleus. (**B**,**C**) Labeling in the longitudinal tori and optic tectum. (**D**) Diffuse and intense labeling over the different layers of optic tectum. (**E**) Diffuse signal probe in the optic tectum, semicircular tori, nucleus of lateral longitudinal fascicle, nucleus of lateral valvula. Abbreviations: CZ, central zone; DWZ, deep white zone; EG, granular eminentiae; Nllf, nucleus of lateral longitudinal fascicle; LV, nucleus of lateral valvula; NG, glomerular nucleus; NRP, nucleus of posterior recess; PGZ, periventricular gray zone; SWGZ, superficial white gray zone; TNp, posterior tuberal nucleus; TS, semicircular tori. Scale bars: (**E**) 100 µm; (**A**–**D**) 200 µm.

In the diencephalon, several positive cells were observed in the dorsal (PPd) and ventral (PPv) periventricular pretectal, anterior and inferior thalamic (A and I), in the ventro-medial (VM) thalamic, and in the magnocellular pre-optic nucleus (PM) nuclei (Figure 6E). The ventro-lateral thalamic nucleus showed numerous intensely stained cells (VL) (Figure 6F). Diffuse positive cells were also observed in the glomerular nucleus (NG), nucleus of posterior recess (NRP), and posterior tuberal nucleus (TNp) (Figure 7A). Several widespread positive cells were also detected in the diffuse lobe of the hypothalamus (DIL) (Figure 6G).

##### Midbrain

Numerous positive cells were seen in the layers of optic tectum, mostly in the periventricular gray zone and in the superficial white zone (Figure 7C,D). Few cells expressing col25a1 mRNA were detected in the longitudinal tori (Tl). In the tegmentum, densely packed positive cells were localized in the semicircular tori (TS), in the nucleus of lateral longitudinal fascicle (Nllf), and in the nucleus of lateral valvula (LV) (Figure 7E).

##### Hindbrain

In the hindbrain, col25a1 mRNA is widely diffused in the granular eminentiae EG, in the granular and molecular layers of the body of the cerebellum (CCe), and in the central griseum (gc).

### 2.4. Characterization of col4a1 and col25a1 Expressing Cells in the Brain of Old Animals

To characterize the cellular phenotypes expressing both col4a1 and col25a1 mRNAs in the brain of old *N. furzeri*, we conducted experiments of in situ hybridization and immunofluorescence via anti-S100β as a marker of glial cell population [32]. We further identified the neuronal phenotype by specifically labeling neuronal processes, neural RNA-binding proteins, and mature neurons via anti-DCX, anti-HuC/HuD, and anti-MAP2, respectively [33].

#### 2.4.1. S100β

**Figure 8:**
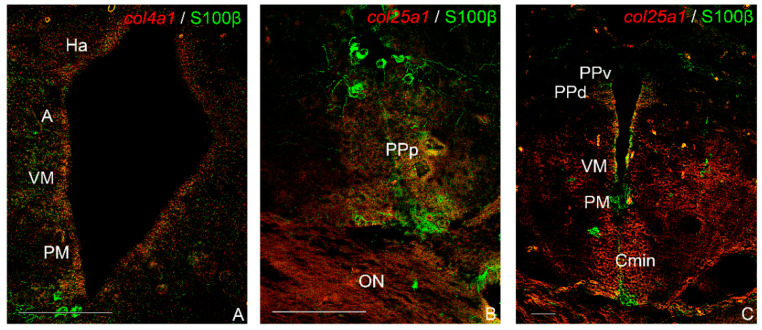
Co-localization of *col4a1* and *col25a1* with anti-S100β in transverse sections of old *N. furzeri* brain. (**A**) *col4a1*/s100β faint co-localization along the diencephalic ventricle. (**B**) Faint *col25a1*/s100β co-localization in in the parvocellular portion of pre-optic nucleus. (**C**) *col25a1*/s100β co-localization along the diencephalic ventricle and in a few sparse glial cells. Abbreviations: A, anterior thalamic nucleus; Cmin, minor commissure; Ha, habenular nucleus; ON, optic nerve; PM, magnocellular pre-optic nucleus; PPd, dorsal periventricular pre-tectal nucleus; PPv, ventral periventricular pre-tectal nucleus; VM, ventro-medial thalamic nucleus. Scale bars: (**A**,**B**) 200 µm; (**C**) 100 µm.

*col4a1*/s100β was faintly co-localized only along the diencephalic ventricle (Figure 8A). In contrast, in the same area, we observed strong co-localization of *col25a1/*s100β and in few sparse thalamic glial cells (Figure 8B,C).

#### 2.4.2. DCX, HuC/HuD, MAP2

**Figure 9:**
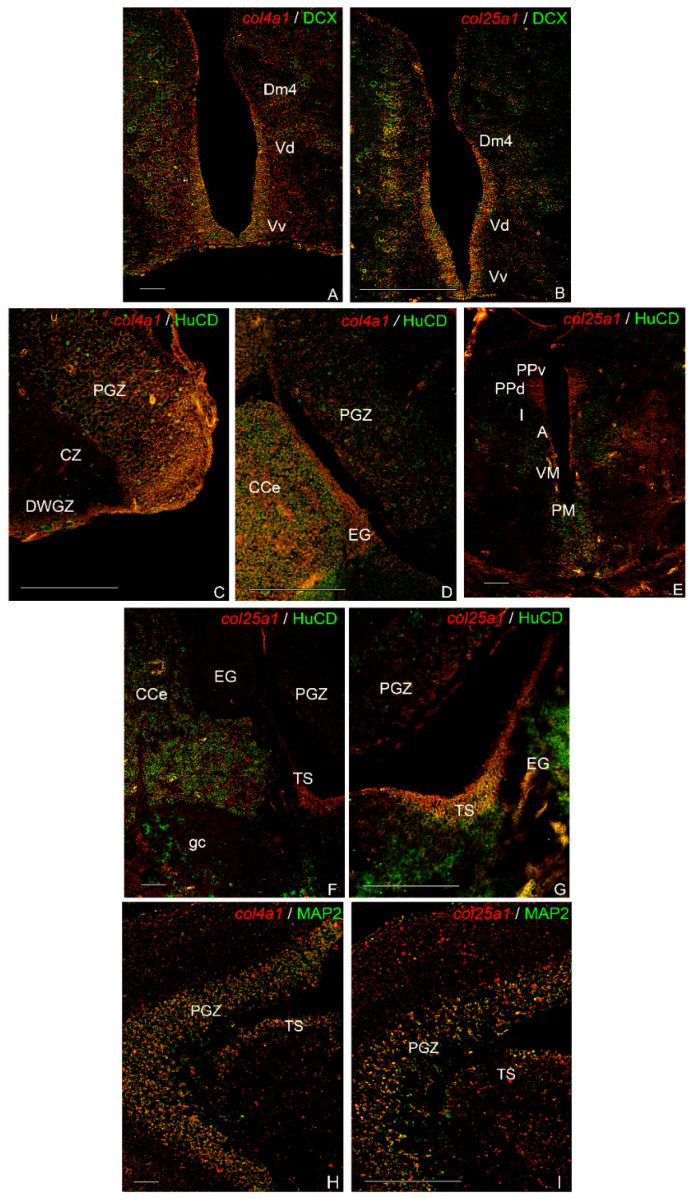
Co-localization of *col4a1* and *col25a1* with anti-DCX, anti-HuC/HuD, and anti-MAP2 in transverse sections of old *N. furzeri* brain. (**A**) Faint *col4a1*/DCX co-staining along the telencephalic ventricle. (**B**) Faint *col25a1*/DCX co-staining along the telencephalic ventricle and in a few neurons of dorsal telencephalon. (**C**,**D**) Weak *col4a1*/HuC/HuD co-localization in the optic tectum, the body of cerebellum and granular eminentiae. (**E**–**G**) Co-localization of *col25a1*/HuC/HuD in very few neurons of magnocellular pre-optic nucleus, in the body of the cerebellum and in the semicircular tori. (**H**) Weak *col4a1*/MAP2 co-localization in a few neurons of the optic tectum. (**I**) Faint *col25a1*/MAP2 co-localization in few neurons of the optic tectum. Abbreviations: A, anterior thalamic nucleus; CCe, body of cerebellum; CZ, central zone of the optic tectum; Dm4, central zone of dorsal telencephalon, layer 4; DWGZ, deep white and gray zone of the optic tectum; EG, granular eminentiae; gc, central griseum; I, inferior thalamic nucleus; PGZ, periventricular gray zone; PM, magnocellular pre-optic nucleus; PPd, dorsal periventricular pre-tectal nucleus; PPv, ventral periventricular pre-tectal nucleus; TS, layer of semicircular tori; Vd, dorsal zone of the ventral telencephalon; VM, ventro-medial thalamic nucleus; Vs, supracommissural zone of ventral telencephalon. Scale bars: (**A**–**G**) 100 µm; (**H**,**I**) 200 µm.

In the forebrain, *col4a1* and *col25a1* were faintly co-localized with DCX along the telencephalic ventricle (Figure 9A,B), whereas *col25a1/*DCX also appeared co-localized in some neurons of layer 4 of the central zone of dorsal telencephalon (Figure 9B). No co-staining was observed in the mid- nor in the hindbrain.

Conversely, *col4a1* and *col25a1* were mainly co-localized with HuC/HuD in the mid- and hindbrain. Weak *col4a1*/HuC/HuD co-localization was observed in the optic tectum, in the PGZ (Figure 9C), in the body of cerebellum and granular eminentiae (EG) (Figure 9D). In very few neurons *col25a1/*HuC/HuD were co-localized in the PM of caudal diencephalon (Figure 9E), in the body of the cerebellum (Figure 9F) as well as in the TS (Figure 9G) and granular eminentiae (EG) (Figure 9G).

Only in a few neurons of the PGZ were *col4a1* and *col25a1* co-localized in MAP2 immunoreactive cells (Figure 9H,I).

## 3. Discussion

In this study, we analyzed the age-dependent regulation of the collagen genes *col4a1* and *col25a1* in the brain of the short-lived teleost *Nothobranchius furzeri*. We showed that both *col4a1* and *col25a1* undergo age-dependent upregulation and that in *N. furzeri*, are expressed in brain region homologous to that of mammals. Furthermore, we showed that both *col4a1* and *col25a1* are expressed in neurons rather than in glial cells.

Collagen genes have been detected in short- and long-lived species [34,35] and their expression was shown to correlate with the lifespan of 33 diverse mammalian species [35]. Ewald and coworkers (2015) confirmed that (i) experimental conditions extending lifespan upregulate collagen genes, and (ii) lifespan extension by genetic reduction in insulin/IGF-1 signaling requires the expression of collagen genes [36]. Based on these results, collagen (and the extracellular matrix in general) undergoes age-dependent modifications and plays an important role in the aging process.

The phylogenetic analysis of *col4a1* and *col25a1* displays a different evolution of the two genes among vertebrate species, with *col4a1* showing a high degree of conservation among vertebrates, even between the short-lived vertebrate *N. furzeri* and humans (61.84% homology). Conversely, the nucleotide sequence of *col25a1* occupies a very distant position compared to the selected actinopterygians or mammalian species.

In agreement with our findings, it has been previously reported that the expression of collagen type IV increases during aging in the human brain, where it accumulates in the basal lamina of cerebral microvessels, therefore, it is considered as a major risk factor for the development of stroke and vascular dementia [9]. Notably, in mouse brain, expression levels of collagen type IV α6 chain followed the expression of Sox2, a key regulator of neurogenesis [37,38]. In the brain of *N. furzeri*, we also observed a parallel age-related increase in *col4a1* and *sox2*, showing that the functional relationship of these two genes might be conserved in this model organism, in which they could possibly be implicated in neuronal physiology (e.g., axon guidance and neurite outgrowth), as already shown in zebrafish [12].

With regard to col25a1 mRNA, this is the first time that *col25a1* has been quantitatively measured in the brain of a vertebrate. A previous study has documented that the expression levels of col25a1 mRNA in the mammalian brain increased from developmental to adult stages [16], and the expression levels remained stable from adult to old stages.

The age-increased levels of the *col4a1* and *col25a1* in the *N. furzeri* brain were confirmed by the wide expression observed via in situ hybridization. In the brain of five week post hatching (wph) animals, col4a1 and col4a1 mRNAs localization was restricted to very few brain areas of the fore- and midbrain, with *col4a1* being expressed only in the optic tectum. The wide neuroanatomical expression patterns of *col4a1* and *col25a1* in old animals appear in several areas that are homologous to the adult mammalian brain [6]. In more detail, in *N. furzeri* col4a1 mRNA is expressed in the (a) olfactory bulbs, the brain region where the olfactory stimulus is integrated first in vertebrates brain; (b) dorsal telencephalic areas homologous to the mammalian hippocampus; and (c) brain areas responsible for integrating visual stimuli such as optic lobes.

A more detailed description of col25a1 mRNA in the adult murine brain reports that it is expressed in the retina and appears specifically enriched in retino-recipient nuclei within the brain (including the suprachiasmatic nucleus, lateral geniculate complex, olivary pretectal nucleus, and superior colliculus) [6]. Notably, in *N. furzeri*, we observed strong *col25a1* labeling in brain nuclei involved in visual processing (i.e., preoptic nuclei, longitudinal tori) and the highest expression in the optic tectum, where the visual stimulus is processed and integrated in fish. These observations contribute to further corroborate the hypothesis that col25a1 is evolutionary conserved in the visual processing systems of vertebrates.

To gain more insights into the cell phenotype expressing *col4a1* and *col25a1*, we employed S100β to identify glial cells [27] and DCX, HuC/HuD, and MAP2 to identify neurons [39] by combined in situ hybridization and immunofluorescence experiments. Very interestingly, *col4a1* and *col25a1* were both expressed in neuronal cells and not in glial cells. Our observations are of valuable interest in the role and expression of collagen genes in the brains of vertebrates, demonstrating the neuronal expression of both genes. However, some differences were observed in the neuronal expression of *col4a1* and *col25a1*: for instance, in the same brain region as caudal telencephalon, we documented the expression of *col25a1* in some DCX immunopositive neurons and very few in the case of *col4a1*/DCX co-labeled neurons. When analyzing *col4a1*/HuC/HuD and *col25a1*/HuC/HuD as well as *col4a1*/MAP2 and *col25a1*/MAP2 co-stainings, very few neurons of mid- and hindbrain appeared co-localized. Interestingly, RNA-Seq analysis coupled with qPCR and in situ hybridization carried out on the cerebral cortex of wild type mouse models, identified different collagen enriched genes and splicing isoforms [40], and clearly confirmed that *col25a1* is synthesized by mature neurons. In contrast, *col4a1* is not expressed in neuronal cell types but only in pericytes [40].

Finally, our observations on the age-dependent changes of *col4a1* and *col25a1* are corroborated by the further characterization of the aging phenotype of the *N. fuzeri* brain. To this aim, we analyzed the age-dependent expression of the major brain cell lineage markers: *pcna*, *dcx*, *gfap*, *s100β*, and *sox2*. We observed no significant differences in the expression of *pcna* between 5 wph and 27 wph animals. Significant upregulation of *pcna* has been previously reported by Terzibasi Tozzini et al. (2012) between 32 wph and 25 wph animals. Our data suggest that this gene might undergo age-related upregulation during later stages, probably after 25–27 wph. We confirmed the upregulation of *gfap* and *s100β* in the brain of 27 wph animals. Both *gfap* and *s100β* are markers of gliosis, and their upregulation during aging has been previously reported in the same model at 32 wph by Terzibasi Tozzini et al. [26]. Our results suggest that the brain aging phenotype already appears around the age of 27 wph. Noteworthy, S100 proteins are reported to be upregulated during aging and/or in the course of brain injuries and neurodegeneration [41]. With regard to neuronal markers, our experiments showed a significant increase in *sox2* (i.e., the sex determining region Y-box 2) in the brain of *N. furzeri* at 27 wph, whereas it is reported to diminish with aging in mouse and human [31]. Unchanged levels of *dcx* expression upon aging was observed when comparing the brain of young (5 wph) and old (27 wph) specimens, unlike what has been reported in the human hippocampus [39]. Altogether, these data open intriguing avenues for future studies on the timing and identification of potential hallmarks of aging in the brain of this model species.

However, we have to consider that the impact of our results could have provided more exhaustive information by including in the study the comparison with another model species such as zebrafish. In addition, the evaluation of the expression of the two target genes at a further time-point, specifically an intermediate age between five and 27 weeks would have provided interesting insights to identify the moment of transition between the non-expression and the expression of the two target genes over aging.

In any case, our findings represent robust evidence that *col4a1* and *col25a1* are evolutionary well conserved in vertebrates, further confirmed by the neuronal expression of *col25a1*, and shed light on the potential neuronal role of *col4a1*. Thus, the age-related increase in the two genes may help to gain more insights into the crucial role of collagen in brain aging onset.

## 4. Materials and Methods

### 4.1. Protocols

The protocols for animal care and use were approved by the appropriate committee at the University of Naples Federico II (2015/0023947). All experimental procedures involving animals were carried out in accordance with the European Parliament and the Council of the European Union Directive of 22 September 2010 (2010/63/UE) and Italian law (D. lgs. 26/2014).

### 4.2. Phylogenetic Analysis and Protein Alignment

Putative *col4a1* (GAIB01100805.1) and *col25a1* (GAIB01090796.1) coding sequences were retrieved from GeneBank-NCBI. Available from: https://www.ncbi.nlm.nih.gov/, accessed on 9 January 2022). The evolutionary history was inferred by using the maximum likelihood (ML) method and Tamura-Nei model [42]. The bootstrap consensus tree inferred from 100 replicates [43] was considered to represent the evolutionary history of the taxa analyzed. Branches corresponding to partitions reproduced in less than 50% bootstrap replicates were collapsed. Initial tree(s) for the heuristic search were obtained automatically by applying neighbor-join and BioNJ algorithms to a matrix of pairwise distances estimated using the maximum composite likelihood (MCL) approach, and then selecting the topology with superior log likelihood value. This analysis involved 28 nucleotide sequences. Codon positions included were 1st + 2nd + 3rd + Noncoding. All positions containing gaps and missing data were eliminated (complete deletion option). There was a total of 2338 positions in the final dataset. Evolutionary analyses were conducted in MEGA X [44].

Protein alignment was performed with the ClustalOmega program (EMBL-EBI, Wellcome Genome Campus, Hinxton, Cambridgeshire, CB10 1SD) [45] for human, mouse, zebrafish, and killifish col4a1 and col25a1 (Supplementary Figure S1).

### 4.3. Animals and Tissue Sampling

All experiments were performed on group-housed *N. furzeri*, wild-type MZM-04/10 strain, of both sexes. Maintenance was performed as previously described [46]. In order to avoid the effect of circadian rhythms and feeding, respectively, animals were suppressed around 10 a.m. and in the fasted state. Young [5 weeks post hatching (wph)] and old (27 wph) animals were anesthetized by immersion in tricaine methanesulfonate (MS-222, Sigma-Aldrich, 300 mg/L of in aqueous solution). For RNA extraction, fish were decapitated, brains were rapidly dissected, kept in sterile tubes (Eppendorf) with 500 µL of RNA later (Qiagen), and stored at 4 °C until RNA extraction. For fluorescence in situ hybridization (FISH) and combined FISH with immunofluorescence (IF), fish were decapitated, heads were rapidly excised and fixed in 4% paraformaldehyde (PFA) in phosphate buffered saline (PBS) treated with diethylpyrocarbonate (DEPC) for 24 h at 4 °C. Brains were then embedded for cryostat sectioning by successive incubation in 30% sucrose solution and 20% sucrose solution at 4 °C overnight (ON). After embedding, samples were frozen at −80 °C. Serial transversal sections of 14 µm thickness were cut with a Leica cryostat (Deerfield, IL).

### 4.4. RNA Isolation and cDNA Synthesis

For RNA extraction, tissues were taken out of RNA later and the excess reagent was removed by means of sterile pipettes. *N. furzeri* total RNA was isolated from 10 animals for each time point (5 wph and 27 wph) with QIAzol (Qiagen), as previously described [44]. Homogenization was performed using a TissueLyzer II (Qiagen) at 20 Hz for 2–3x 1 min. Total RNA was then quantitated with Eppendorf BioPhotometer and 500 ng was retrotranscribed to cDNA using the QuantiTect^®^ Reverse Transcription Kit (Qiagen) and following the supplier’s protocol. The newly synthetized cDNA was then diluted to a final volume of 200 µL with ultra-pure sterile nuclease free water to an approximate final cDNA concentration of 40 ng/µL.

### 4.5. Identification of col4a1 and col25a1

According to the sequence information (Nofu_GRZ_cDNA_3_0012842), one set of primers was designed to amplify *N. furzeri* col4a1 and col25a1 cDNAs (*col4a1* forward 5′-GGATTCCCTGGAGAAAAAGG-3′; col4a1 reverse 5′-ACAGCTTCCTGCCGTACCTA-3′; *col25a1* forward 5′-TGCTTCAAACCCCTCCCTCTT-3′; *col25a1* reverse 5′-TGTATCAGAGGCTGCGAGA-3′). Primers were designed to amplify a fragment of 465 base pairs (bp) and 390 bp, respectively, for *col4a1* and *col25a1*. PCR reactions were carried out with Phusion^®^ High-Fidelity PCR Master Mix (New England BioLabs^®^ Inc., Ipswich, MA, USA) following the manufacturer’s instructions. Cycling conditions were as follows: 94 °C for 3 min, 94 °C for 30 s (35 cycles), 54° C for 30 s, and 72 °C for 1 min. An additional final extension step at 72 °C for 7 min was added. PCR products were resolved on 2% agarose gel at 120 V for 50 min. DNA was precipitated with ethanol and quantified. A total of 300 ng of DNA were sequenced at the Leibniz Institute on Aging, Fritz Lipmann Institute laboratories (Jena, Germany). Sequences thus obtained were compared and matched with the information available on NFINtb *N. furzeri* transcriptome browser (http://nfintb.leibniz-fli.de/nfintb/, accessed on 9 January 2022).

### 4.6. Phylogenetic Analysis and Protein Alignment

col4a1 and col25a1 cDNA translated sequences were used as queries to recover orthologues from Genbank-NCBI (National Center for Biotechnology Information (NCBI) [Internet]. Bethesda (MD): National Library of Medicine (USA), National Center for Biotechnology Information; [1988]—[cited 18 June 2020]. Available from: https://www.ncbi.nlm.nih.gov/, accessed on 9 January 2022). Phylogenetic analysis was conducted with MEGA X program, using the following sequences: Poecilia reticulata (*col4a1* XM_008401564.2, col25a1 XM_008435849.2), *Haplochromis burtoni* (*col4a1* XM_005948835.2 and *col25a1* XM_005927689.2), *Amphirion ocellaris* (*col4a1* XM_023267280.1 and *col25a1* XM_023299956.1), *Poecilia formosa* (*col4a1* XM_007555367.2 and *col25a1* XM_016675245.1), *Cynoglossus semilaevis* (*col4a1* XM_008328686.3 and *col25a1* XM_025062607.1), *Kryptolebias marmoratus* (*col4a1* XM_017410233.2 and *col25a1* XM_017414412.1), *Oryzias latipes* (*col4a1* NM_001177472.1 and *col25a1* XM_020711604.2), *Stegastes partitus* (*col4a1* XM_008275532.1 and *col25a1* XM_008294251.1), *Xiphophorus maculatus* (*col4a1* XM_005798133.2 and *col25a1* XM_023346157.1), and *Oryzias melastigma* (*col4a1* XM_024286964.1 and *col25a1* XM_024288338.1).

Evolutionary history was inferred using the maximum likelihood method [40]. *Homo sapiens* (*col4a1* GenBank NM_001845.6, *col25a1* GenBank NM_198721.4), *Mus musculus* (*col4a1* GenBank NM_009931.2, *col25a1* GenBank NM_029838.4), and *Danio rerio* (*col4a1* GenBank XM_688948.9, *col25a1* GenBank XM_009303545.3) mRNA sequences were also included.

Protein sequence alignment for human (*col4a1* GenBank AAI51221.1, *col25a1* GenBank AAH36669.1), mouse (*col4a1* GenBank AAH72650.1, *col25a1* GenBank AAI38052.1), zebrafish (*col4a1* GenBank XP_694040.5, *col25a1* GenBank XP_009301813.1), and turquoise killifish (*col4a1* GenBank SBP39483.1, *col25a1* GenBank XP_015801986.1) was also performed. For aminoacidic analysis, the Clustal W program was employed [44].

### 4.7. Quantitative Real Time-PCR (qPCR)

qPCR experiments were performed to evaluate the expression levels of the following molecular markers: *pcna* (proliferating cell nuclear antigen) strictly confined to proliferating cells), *dcx* (doublecortin), *sox2* (sex-determining region Y box 2) [29], *s100β* and *gfap* [27], and *col4a1* and *col25a1* mRNAs in the brain of young and old animals. Primers were designed with the Primer3 tool [47]. According to the sequence information, one set of primers was designed to quantize each cDNA (Table 1). *N. furzeri* primers for *pcna* and *gfap* were retrieved from [27]. Reactions were performed in a 20 µL volume containing 1 µL of diluted cDNA, 10 µL of BrightGreen 2× qPCR MasterMix, 0.6 µL of each 10 µLM primer, free nuclease water up to 20 µL using the BrightGreen 2× qPCR MasterMix Kit (abm^®^) following the manufacturer’s instructions. Reactions were performed in triplicate and negative control (water) was always included. TATA box binding protein (TBP) mRNA was included as the housekeeping gene. Experiments were run on a 7300 Real-Time PCR System (Applied Biosystems).

### 4.8. Statistical Analysis

Expression levels of col4a1, col25a1, pcna, dcx, sox2, s100β, and gfap mRNAs were analyzed by the ΔΔCt method and normalized to the expression of the reference gene TBP ± SEM. Differences in expression levels for each time point (5 wph and 27 wph) were measured through fold change (FC) values to TBP (log2 base scale). The results in output were subsequently employed to build the relative ΔΔCt. Graphic was built with the ggplot2 package [48] for R programming language (R Development Core Team, 2008).

### 4.9. Riboprobe Synthesis

Riboprobes to identify *N. furzeri* col4a1 and col25a1 mRNA were synthesized by in vitro transcription (IVT) using the MAXIscript ™ SP6/T7 IVT Kit (Invitrogen by Thermo Fisher Scientific–Catalogue number AM1312) following the manufacturer’s instructions. One µg of DNA template was transcribed to RNA in 20 µL volume reaction including 5 µL of 10× DIG RNA labeling mix (Roche, cat. 11 277 073 910) containing 3.5 mM of digoxigenin labeled uracil (DIG-11-UTP). T7 RNA polymerase was employed. Therefore, the T7 promoter sequence (5′-GGTAATACGACTCACTATAGG-3′) was associated upstream to the reverse primers of *col4a1* and *col25a1*. All components were briefly centrifuged and incubated at 37 °C for 1 h. Then, 1 µL of turbo DNase 1 was added, the sample was mixed well, and incubated for 15 min at 37 °C. One µL of EDTA 0.5 M, pH 8.8, was added to stop the reaction. Reaction products were analyzed by gel electrophoresis and quantified. To validate and determine the riboprobe concentration, the dot blot technique was employed. Riboprobe’s fresh dilution buffer was prepared mixing DEPC H2O, saline sodium citrate buffer (SSC) 20×, and formaldehyde 37% (5:3:2). Probes were then diluted in the dilution buffer, as indicated in Table 2, in a final volume of 20 µL. A negative control was included for each probe. One µL of each probe dilution plus negative controls were then pipetted onto a nitrocellulose membrane, forming little separated spots (Table 1). The membrane was then fixed in the Stratalinker^®^ UV Crosslinker for 30 s, gently washed in 1 × PBS and incubated for 30 min with in situ blocking solution (BS) (1 × PBS, 10% of sheep serum heat inactivated, 0.5% Roche blocking reagent, 0.1% Tween-20, DEPC H_2_O to final volume). Riboprobes were then incubated in anti-digoxigenin-AP, Fab fragments from sheep (Roche, Germany), 1:2000 in BS. Subsequently, the membrane was washed twice for 15 min in 1 × PBS and incubated with a BM purple chromogenic substrate for alkaline phosphatase (AP) (Merck, Darmstadt, Germany) until detection of the mRNA’s spots. A final concentration of 500 pg/µL was taken into account.

### 4.10. Fluorescence In Situ Hybridization

Fluorescence in situ hybridization (FISH) experiments were conducted on cryostat sections by means of sterile solutions and materials. DEPC was added to PBS and water 1 mL/L to inactivate RNase enzymes; solutions were shaken vigorously and autoclaved.

Sections were dried for 2 h at room temperature (RT), well washed in 1 × DEPC/PBS and treated with 10 µg/µL Proteinase K (Sigma–Aldrich, St. Louis, MO, USA) 1:200 in 1 × PBS for 10 min. Proteinase K action was then inactivated by two washes in 2 mg/mL glycine, 5 min each. Sections were post fixed in 4% PFA for 20 min and well washed in 1 × PBS at RT. A pre-hybridization step was carried out in the hybridization solution (HB) (50% formamide, 25% 20 × SSC, 50 µg/mL heparin, 10 µg/mL yeast RNA, 0.1% Tween 20, 0.92% citric acid) at 55 °C for 1 h. Riboprobes were then denatured for 10 min at 80 °C, diluted to a final concentration of 500 pg/µL in HB and employed to hybridize sections, ON at 52 °C. Post-hybridization washes were carried out at 55 °C as follows: 2 × 20 min in 1 × SSC, 2 × 10 min in 0.5 × SSC, 1 × 5 min 2 × PBS at RT. Sections were blocked in BS for 1 h at RT. Afterward, sections were incubated in anti-digoxigenin-AP, Fab fragments from sheep (Roche, Germany), 1:2000 in BS for 2 h at RT. Sections were well washed in 1 × PBS. Chromogenic reaction was carried out by using Fast Red tablets (Sigma-Aldrich, St. Louis, MO, USA) in Tris buffer and incubating the slides at RT, in the dark. Signal was observed every 20 min until signal detection. After signal developing, sections were washed in 1 × PBS at RT and mounted with Fluoreshield Mounting Medium with DAPI as counterstaining for the nuclei.

### 4.11. Combined FISH with IF

After the detection of FISH chromogenic reaction, sections were well washed in 1 × PBS and incubated at RT for 1 h with blocking serum (normal goat serum 1:5 in 1 × PBS containing 0.1% Triton X-100 from Sigma, St. Louis, MO, USA) and subsequently with primary antisera against different neural markers (Table 3), ON at 4 °C. Sections were then well washed in 1 × PBS and incubated with secondary antibodies: goat anti-rabbit IgG (H+L) Alexa fluor™ Plus 488 (Invitrogen by Thermo Fisher Scientific, Waltham, MA, USA, ref A32731), goat anti-mouse IgG (H+L) Alexa fluor™ Plus 488 (Invitrogen by Thermo Fisher Scientific, ref A32723), 1:1000 in PBS/DEPC, and Alexa Fluor 488–conjugated Streptavidin, 1:500 (Jackson Immunoresearch, ref 016–540–084).

### 4.12. Image Acquisition and Processing

FISH reactions were observed and analyzed with a Zeiss AxioScope AX 1.0 microscope (Carl Zeiss, Jena, Germany) with AxioCam MC5 and AxioVision software and Leica–DM6B (Leica, Wetzlar, Germany) software. Double FISH/IHC reactions were observed and analyzed with Leica–DM6B (Leica, Wetzlar, Germany) and LasX software. Digital raw images were optimized for image resolution, contrast, evenness of illumination, and background using Adobe Photoshop CC 2018 (Adobe Systems, San Jose, CA, USA). Anatomical structures were identified according to the adult *N. furzeri* brain atlas [49].

## 5. Conclusions

This manuscript reports the age-related analysis of two collagen genes, *col4a1* and *col25a1*, in the brain of the short-lived teleost *N. furzeri*. We discovered that age affects both the quantitative expression and neuroanatomical distribution of the genes, with a higher and wider expression in old brains. In depth analysis of the phenotype of cells expressing *col4a1* and *col25a1* mRNAs in the brains of old animals showed that they were preferentially expressed in the neuronal lineage (DCX, HuC/HuD, MAP2 immunoreactive cells).

Our work may stimulate further investigation on the role of *col4a1* and *col25a1* in the onset of neurodegenerative diseases, and more in general in the aging process. Additionally, by further characterizing the aging phenotype of killifish brain, we contribute by adding new features in terms of similarities among killifish and mammals (e.g., structural conservation of genes and protein sequences; conservation of gene regulation, function and distribution), thus enhancing the reliability of *N. furzeri* as a translational model.

Future studies may address the investigation of col4a1 and col25a1 protein distribution and molecular pathways to generate Alzheimer’s or Parkinson’s disease models. These may be extremely useful in various research fields, from drug discovery to nanomedicine, engaged in the development of therapies for the treatment of neurodegenerative diseases.

## Figures and Tables

**Table 1:** Primers employed for qPCR experiments.

Gene	Primer	Sequence (5′-3′)
*dcx*	Forward	AGGTGCTCACTGACATCACA
*dcx*	Reverse	CGCCGAAGAAATCCTGAAGG
*S100β*	Forward	GCGTGTCTACTTGTGTGCAT
*S100β*	Reverse	TTCTCGCCATCTCTCTCGTC
*sox2*	Forward	GAACGGCACCAACCAGAAAA
*sox2*	Reverse	TCGGAGTTGTGCATTTTGGG
*col4a1*	Forward	CTGGAATCCCTGGAGAGCC
*col4a1*	Reverse	CCTGGAGGCCTTGTGTACC
*col25a1*	Forward	AACCTGAAACGCATGCAGTT
*col25a1*	Reverse	TCACGGCAGGACACTCAG
*TBP*	Forward	CGGTTGGAGGGTTTAGTCCT
*TBP*	Reverse	GCAAGACGATTCTGGGTTTG

**Table 2:** Serial dilution for col4a1 and col25a1 mRNA probes employed for dot blot validation.

Dilution Number	Final Concentration
1	2 ng/µL
2	500 pg/µL
3	100 pg/µL
4	50 pg/µL
5	25 pg/µL
6	10 pg/µL
7	5 pg/µL
8	2.5 pg/µL
9	1 pg/µL
10	0.1 pg/µL

**Table 3:** List of primary antisera employed for combined FISH/IF.

Primary Antiserum	Source	Host	Dilution
anti-S100β	Agilent Dako Z0311	Rabbit Polyclonal	1:200
anti-DCX	Abcam (18723)	Rabbit polyclonal	1:100
anti-HuC/HuD	Invitrogen by Thermo Fisher Scientific (A21272)	Mouse IgG2b, biotin-XX conjugate	1:50
anti-MAP2	Santa Cruz Biotechnology (A8): sc-74422	Mouse Monoclonal	1:100

## Data Availability

Not applicable.

## Supplementary Materials

The following supporting information can be downloaded at: https://www.mdpi.com/article/10.3390/ijms23031778/s1.

## Abbreviations

MDPI	Multidisciplinary Digital Publishing Institute
DOAJ	Directory of open access journals
TLA	Three letter acronym
LD	linear dichroism
